# Improving Hip Fracture Prediction by Using Sarcopenia‐Specific Cut‐Offs of T‐Scores for Osteosarcopenia: A Prospective Cohort Study

**DOI:** 10.1002/jcsm.70348

**Published:** 2026-07-15

**Authors:** Anjo Zhihui Lu, Freddy Man Hin Lam, Jason Leung, T. W. Auyeung, Jenny Lee, King‐Son Lai, Jean Woo, Timothy Kwok

**Affiliations:** ^1^ School of Nursing The Hong Kong Polytechnic University Hong Kong China; ^2^ Department of Rehabilitation Sciences The Hong Kong Polytechnic University Hong Kong China; ^3^ Jockey Club Centre for Osteoporosis Care and Control The Chinese University of Hong Kong Hong Kong China; ^4^ Jockey Club Institute of Ageing The Chinese University of Hong Kong Hong Kong China; ^5^ Department of Medicine and Geriatrics Tai Po Hospital Hong Kong China; ^6^ Department of Medicine and Therapeutics The Chinese University of Hong Kong Hong Kong China

**Keywords:** ageing, bone mass, fractures, muscle strength, osteoporosis, osteosarcopenia, sarcopenia

## Abstract

**Background:**

Older adults with osteosarcopenia, defined as the coexistence of osteopenia/osteoporosis and sarcopenia, have been associated with an increased risk of fractures compared with those with normal bone mineral density (BMD) and without sarcopenia. However, the conventional definition of osteosarcopenia has led to mixed results on its predictive accuracy for hip fractures. We aimed to define osteosarcopenia by identifying sex‐specific cut‐offs for low BMD according to different sarcopenia statuses and compare its predictive performance with osteosarcopenia defined conventionally and osteoporosis alone.

**Methods:**

A cohort of 4000 community‐dwelling older adults (2000 females, mean age 72.5 ± 5.2 years) was recruited. Body composition and BMD of hip, lumbar spine and femoral neck were measured using dual energy X‐ray absorptiometry at baseline. According to the Asian Working Group for Sarcopenia 2019 consensus, all participants were classified as non‐sarcopenia, possible sarcopenia and sarcopenia. Incidence of hip fractures was documented during the follow‐up period from 2001 to 2013. The classification and regression tree (CART) analysis was performed to identify the optimal cut‐off values of T‐scores for participants with possible sarcopenia and those with sarcopenia, respectively. Cox proportional hazards regression models were used to estimate the associations between osteosarcopenia and hip fractures. The discrimination and predictive ability of osteosarcopenia were evaluated by Uno's concordance index (C‐index), time‐dependent net reclassification improvement (NRI) and integrated discrimination improvement (IDI).

**Results:**

During an average of 9.2 years of follow‐up, 63 (3.2%) men and 69 (3.5%) women had at least one hip fracture. Sex‐ and sarcopenia‐specific cut‐offs of T‐scores were identified (men with possible sarcopenia: T‐score < −2.2; men with sarcopenia: T‐score < −2.0; women with possible sarcopenia: T‐score < −2.1; women with sarcopenia: T‐score ≤ −2.5). Osteosarcopenia defined with these specific cut‐offs was associated with an increased risk of hip fractures independent of clinical risk factors in both men and women (HR = 5.232, 95% CI = 3.172–8.631, and HR = 3.574, 95% CI = 2.01–6.355, respectively). It had the highest Uno's C‐index (men: 0.774 and women: 0.750, respectively) and outperformed osteosarcopenia using conventional definition and osteoporosis alone in predicting hip fractures by using NRI and IDI approaches.

**Conclusions:**

Our findings suggested that including possible sarcopenia and adopting sex‐ and sarcopenia‐specific cut‐offs to define osteosarcopenia could improve hip fracture prediction. Further studies are warranted to validate the cut‐offs of T‐score identified in our cohort.

## 1 Introduction

As the population ages, hip fractures have always been an important public health issue as they can lead to morbidity and a significant burden to the medical and social services [1]. Low bone mineral density (BMD) contributes significantly to hip fractures [2], while sarcopenia, which leads to loss of muscle mass and muscle strength, could also predispose older adults to a higher risk of falls and fractures [3]. Together, low BMD (osteopenia: T‐score between −2.5 and −1; or osteoporosis: T‐score ≤ −2.5) and sarcopenia form a newly established geriatric syndrome known as ‘osteosarcopenia’, which is associated with increased risk of falls, fractures and hospitalizations [4].

Although it has been acknowledged that individuals with osteosarcopenia were at a greater risk of fractures than those without osteosarcopenia [4], it remains controversial whether osteosarcopenia could provide greater predictive power for fractures over low BMD alone—the well‐established predictor of fractures [2]. Several studies found that individuals with osteosarcopenia have a greater risk of fractures as compared with those with only osteopenia or osteoporosis [5, 6, 7], whereas other studies did not [8, 9, 10, 11]. These discrepancies could be due to the sex difference and the non‐unified definition of osteosarcopenia [12]. The incremental predictive value of osteosarcopenia over low BMD alone was predominantly reported among older men but not older women [5, 6, 7]. Furthermore, including osteopenia in the definition of osteosarcopenia has shown inconsistent additional predictive value for fractures [8, 9, 10, 11]. Some studies found that sarcopenia combined with osteoporosis but not osteopenia improved fracture prediction [9, 11]. This suggests that a more precise cut‐off for low BMD rather than simply categorizing people as osteopenia or osteoporosis to define osteosarcopenia is required to better identify individuals at risk of hip fractures.

Sarcopenia status, the other key component of osteosarcopenia, is closely related to BMD because of the muscle–bone crosstalk [13]. Previous studies demonstrated that individuals with sarcopenia had lower BMD and poorer bone architecture than those with no sarcopenia [14, 15, 16]. Individuals who had low muscle strength but normal muscle mass also had a significantly poorer bone structure when compared with those with no sarcopenia [15]. Hence, the threshold of BMD to identify individuals with higher risk of fractures might vary across different sarcopenia statuses.

In addition, the decrease in muscle strength and physical performance has been found to be important predictors of falls [17], which could in turn lead to a higher risk of fractures. Indeed, previous studies including ours have demonstrated that in the absence of muscle mass, low BMD combined with low muscle strength and/or low physical performance were associated with a greater risk of fractures [6, 7, 10, 18]. Muscle strength, but not muscle mass, was observed to be associated with an increasing risk of falls and fractures over 10 years in a previous study [19]. Unfortunately, the current definition of osteosarcopenia only includes sarcopenia but not possible sarcopenia, which is defined as low muscle strength or low physical performance only [20]. It is therefore essential to evaluate whether taking possible sarcopenia into consideration in defining osteosarcopenia could improve its hip fractures prediction.

Given that the current definition of osteosarcopenia may have hampered its predictive value in fractures, this prospective cohort study of community‐dwelling Chinese older adults including people with possible sarcopenia and confirmed sarcopenia has the following aims: first, to identify sex‐specific cut‐off points of BMD T‐score according to the sarcopenia status (i.e., possible sarcopenia and sarcopenia) and to update the definition of osteosarcopenia; and second, to evaluate the hip fracture prediction ability of osteosarcopenia using the updated definition and compare it with osteoporosis and osteosarcopenia using the conventional definition. We hypothesized that osteosarcopenia defined with the sex‐ and sarcopenia‐specific cut‐off points of BMD T‐scores could improve the prediction of hip fractures over osteoporosis and osteosarcopenia defined conventionally, and thus, more individuals with osteosarcopenia at risk of hip fractures could be identified for fracture prevention or potential interventions.

## 2 Methods

### 2.1 Participants

Participants were recruited from the Mr. OS and Ms. OS Hong Kong cohort study, a prospective cohort study that consisted of 4000 community‐dwelling men and women aged 65 years and older in Hong Kong between August 2001 and December 2003. The participants were mainly recruited by posting advertisements in housing estates and local community centres. Those who were unable to walk independently, had bilateral hip replacement or were not able to give informed consent were excluded. The sample was age‐stratified so that approximately equal numbers of participants were in the age range of 65 to 69, 70 to 74 and 75 years and older.

### 2.2 BMD

BMD of total hip, lumbar spine (L_1_–L_4_) and femoral neck were measured by Hologic QDR 4500‐W densitometers (Hologic Inc., Waltham, MA, USA). The coefficients of variation (in vivo) were 0.7% for the total hip BMD, 0.9% for the lumbar spine BMD and 1.3% for the femoral neck BMD. The World Health Organization defines osteopenia and osteoporosis via BMD T‐score for total hip or/and lumbar spine or/and femoral neck: (1) osteopenia −1.0 to −2.5 standard deviation and (2) osteoporosis: ≤ −2.5 standard deviation.

### 2.3 Sarcopenia

Sarcopenia status was defined according to the Asian Working Group for Sarcopenia 2019 consensus (AWGS 2019) [20]. In this study, whole and regional body composition was measured with the same densitometer at the same time as the BMD measurement. Appendicular skeletal muscle mass (ASM) was calculated as the sum of appendicular lean mass minus bone mineral content of both arms and legs, with the operator adjusting the cut lines of the limbs according to specific anatomical landmarks as described by Heymsfield et al. [21]. Body height was measured by Holtain Harpenden stadiometer (Holtain Ltd) for the calculation of height‐adjusted ASM. Grip strength was measured using a dynamometer (JAMAR Hand Dynamometer 5030J1; Sammons Preston). Two readings were taken from each side, and the maximum value of the four readings was used for analysis. Gait speed was measured by asking the participant to complete a 6‐m walkway at usual pace for twice, and the best time in seconds was used. The five‐time chair‐stand test was measured by the time taken to complete five repeated chair stands without using arms as quickly as possible. As suggested by AWGS 2019, low muscle mass was defined as ASM index (ASM/height^2^) < 7.0 kg/m^2^ for men and < 5.4 kg/m^2^ for women; low muscle strength was defined as grip strength < 28 kg for men and < 18 kg for women; and low physical performance as gait speed < 1.0 m/s or five‐time chair‐stand tests ≥ 12 s. Sarcopenia was defined as a person who has low muscle mass, low muscle strength and/or low physical performance. In addition, among participants without low muscle mass, those with low muscle strength and/or low physical performance were categorized as possible sarcopenia. Therefore, all participants were categorized as non‐sarcopenia, possible sarcopenia and sarcopenia.

### 2.4 Covariates

The clinical risk factors in Fracture Risk Assessment Tool (FRAX) including age, sex, BMI, previous history of fracture, parental history of hip fracture, current smoking, at least three alcoholic beverages per day, glucocorticoids use, rheumatoid arthritis, secondary osteoporosis and femoral neck BMD were collected. The WHO 10‐year absolute risk of hip fracture (FRAX score) was calculated by the WHO Collaborating Centre for Metabolic Bone Disease, using the FRAX algorithm (Hong Kong version) [22]. The 10‐year probability of hip fracture calculated without femoral neck BMD was calculated. In addition, history of falls in the past 12 months, physical activity assessed using the validated Physical Activity Scale for the Elderly (PASE) Questionnaire [23] and number of diseases (categorized as 0, 1–2 and ≥ 3) were obtained through a questionnaire interview.

### 2.5 Ascertainment of Incident Fracture (Outcome Measures)

Incident hip fractures were documented for up to 10 years or more (2001–2013). All participants were followed up for hip fracture incidence by visits to the research centre at two yearly intervals for the first 6 years, and telephone calls every 4 months in the first 4 years. This was supplemented and verified by carrying out a search of the territory‐wide electronic medical record system of the Hospital Authority of Hong Kong, which includes all visits to the Accident and Emergency Departments and outpatient clinics and covers over 95% of all hospital admissions in Hong Kong.

### 2.6 Statistical Analysis

The baseline characteristics of participants with different sarcopenia status (non‐sarcopenia, possible sarcopenia and sarcopenia) were compared using one‐way ANOVA or chi‐square tests as appropriate. To identify the cut‐off values of T‐scores for low BMD according to different sarcopenia statuses, the classification and regression tree (CART) analysis with 10‐fold cross‐validation was performed because the cut‐off points are chosen by optimizing discrimination of the outcome in CART analysis [24]. The lowest value of BMD T‐score of total hip, lumbar spine and femoral neck was used in CART analysis, and the optimal cut‐offs of T‐score in different sarcopenia status were identified for hip fracture prediction. Conventionally, osteosarcopenia is defined as the coexistence of osteopenia/osteoporosis and sarcopenia (Osteosarcopenia_conventional_). In this study, we proposed to define osteosarcopenia based on the sex‐specific cut‐off points of T‐score according to different sarcopenia status identified by CART analysis (osteosarcopenia_CART_).

Cox proportional hazards regression models were used to estimate the associations of osteoporosis, osteosarcopenia_conventional_ and osteosarcopenia_CART_ with time to incident hip fractures. The proportional hazards assumption was confirmed using log‐minus‐log survival plots, and no collinearity was detected based on the variance inflation factor (VIF). Hazard ratios (HRs) with 95% confidence intervals (95% CI) were reported. To evaluate whether the associations were independent of other common risk factors of fractures, FRAX hip fracture risk scores were further controlled in the subsequent models. To assess the discrimination ability of the models, Uno's concordance index (C‐index) was calculated and compared between models, providing a robust measure for censored data [25]. Additionally, reclassification improvement was further evaluated using the time‐dependent net reclassification improvement (NRI) index and the integrated discrimination improvement (IDI) index [26].

Several sensitivity analyses were conducted. First, to evaluate the robustness of the associations between osteosarcopenia_CART_ and risk of hip fractures, the Cox regression models were repeated with adjustment of (1) FRAX score and other risk factors of fractures including history of falls, physical activity and numbers of diseases; and (2) individual components of FRAX. Next, the main analyses were repeated in two subsamples: (1) those without osteoporosis medication and (2) those who were classified as non‐sarcopenia and possible sarcopenia.

CART analysis was performed using the r‐part procedure of R (Version 4.3.3), and the other analyses were conducted using the Statistical Analysis System (SAS) version 9.4 (SAS institute Inc., Cary, NC, USA). All tests were two‐sided, and *p* values less than 0.05 were considered statistically significant.

## 3 Results

### 3.1 Characteristics of Participants

During an average of 9.2 years of follow‐up on 4000 older adults (2000 female, mean age: 72.5 ± 5.2 years), 63 (3.2%) men and 69 (3.5%) women had at least one hip fracture. Using the AWGS 2019 algorithm, 760 (38%) men and 1231 (61.6%) women were identified as having possible sarcopenia, while 572 (28.6%) men and 267 (13.4%) women were identified as sarcopenia. The baseline characteristics of the participants according to their sarcopenia status are shown in Table 1. When compared with participants without sarcopenia, sarcopenic men and women were significantly older and had lower BMI, higher FRAX scores and lower BMD at hip, spine and femoral neck. The hip fracture rate during follow‐up was the highest among men and women with sarcopenia, followed by those with possible sarcopenia and then those without sarcopenia.

**TABLE 1:** Characteristics of participants according to sarcopenia status (*n* = 4000).

	Men				Women			
	Non‐sarcopenia (*n* = 668)	Possible sarcopenia (*n* = 760)	Sarcopenia (*n* = 572)	*p*	Non‐sarcopenia (*n* = 502)	Possible sarcopenia (*n* = 1231)	Sarcopenia (*n* = 267)	*p*
Components of sarcopenia								
Grip strength (kg)	36.8 ± 5.7	34.1 ± 6.5	30.3 ± 6.4	< 0.001	24.2 ± 3.8	22.1 ± 4.5	19.9 ± 3.6	< 0.001
Walking speed (m/s)	1.23 ± 0.17	0.99 ± 0.2	0.97 ± 0.21	< 0.001	1.17 ± 0.13	0.88 ± 0.19	0.9 ± 0.18	< 0.001
5‐time chair stand (s)	9.67 ± 1.53	14.19 ± 3.62	14.13 ± 4.15	< 0.001	9.66 ± 1.48	14.89 ± 5.46	13.88 ± 3.49	< 0.001
Appendicular lean muscle mass (kg/m^2^)	7.32 ± 0.81	7.7 ± 0.54	6.39 ± 0.46	< 0.001	6.04 ± 0.69	6.28 ± 0.63	5.07 ± 0.26	< 0.001
Components of FRAX								
Age	70.6 ± 4.2	72.6 ± 4.6	74.2 ± 5.6	< 0.001	70.6 ± 4.6	73 ± 5.3	74.2 ± 5.9	< 0.001
BMI (kg/m^2^)	23.6 ± 3.1	25.1 ± 2.5	21 ± 2.4	< 0.001	23.6 ± 3.3	24.8 ± 3.2	20.6 ± 2.5	< 0.001
Previous history of fracture	96 (14.4%)	99 (13%)	79 (13.8%)	0.759	106 (21.1%)	258 (21%)	52 (19.5%)	0.846
Parental history of hip fracture	25 (4.5%)	37 (5.9%)	21 (4.4%)	0.400	33 (7.6%)	44 (4.4%)	10 (5%)	0.041
Current smokers	83 (12.4%)	66 (8.7%)	89 (15.6%)	< 0.001	9 (1.8%)	20 (1.6%)	8 (3%)	0.295
≥ 3 alcoholic beverages per day	4 (0.6%)	8 (1.1%)	4 (0.7%)	0.599	0 (0.0)	0 (0.0)	0 (0.0)	—
Glucocorticoids use	4 (0.6%)	5 (0.7%)	2 (0.3%)	0.805	1 (0.2%)	3 (0.2%)	1 (0.4%)	0.820
History of rheumatoid arthritis	6 (0.9%)	10 (1.3%)	5 (0.9%)	0.659	2 (0.4%)	28 (2.3%)	8 (3%)	0.013
Secondary osteoporosis	0 (0.0)	0 (0.0)	0 (0.0)	—	0 (0.0)	0 (0.0)	0 (0.0)	—
FRAX scores								
Hip fracture without BMD	2.83 ± 2.09	3.05 ± 2.07	4.68 ± 3.05	< 0.001	5.52 ± 4.97	6.14 ± 4.46	9.77 ± 6.93	< 0.001
Other risk factors of fractures								
History of falls	94 (14.1%)	122 (16.1%)	91 (15.9%)	0.531	110 (21.9%)	303 (24.6%)	69 (25.8%)	0.380
PASE scores	106.1 ± 53.4	97.7 ± 49.5	86.4 ± 45.4	< 0.001	91.2 ± 32.6	85.2 ± 33.6	75.3 ± 29.5	< 0.001
Number of diseases				< 0.001				< 0.001
0	137 (20.5%)	107 (14.1%)	101 (17.7%)		100 (19.9%)	188 (15.3%)	37 (13.9%)	
1–2	363 (54.3%)	375 (49.3%)	299 (52.3%)		285 (56.8%)	619 (50.3%)	164 (61.4%)	
3+	168 (25.1%)	278 (36.6%)	172 (30.1%)		117 (23.3%)	424 (34.4%)	66 (24.7%)	
BMD								
Hip BMD (g/cm^2^)	0.88 ± 0.12	0.89 ± 0.12	0.8 ± 0.13	< 0.001	0.72 ± 0.11	0.71 ± 0.12	0.66 ± 0.11	< 0.001
Spine BMD (g/cm^2^)	0.95 ± 0.17	0.99 ± 0.18	0.9 ± 0.18	< 0.001	0.76 ± 0.15	0.76 ± 0.15	0.7 ± 0.14	< 0.001
Femoral neck BMD (g/cm^2^)	0.7 ± 0.11	0.71 ± 0.11	0.64 ± 0.11	< 0.001	0.59 ± 0.1	0.59 ± 0.1	0.54 ± 0.1	< 0.001
Hip fractures								
Hip fracture in 10 years	9 (1.3%)	24 (3.2%)	30 (5.2%)	< 0.001	7 (1.4%)	46 (3.7%)	16 (6%)	0.003

Abbreviations: BMD, bone mineral density; BMI, body mass index.

### 3.2 Updated Definition of Osteosarcopenia

**FIGURE 1:**
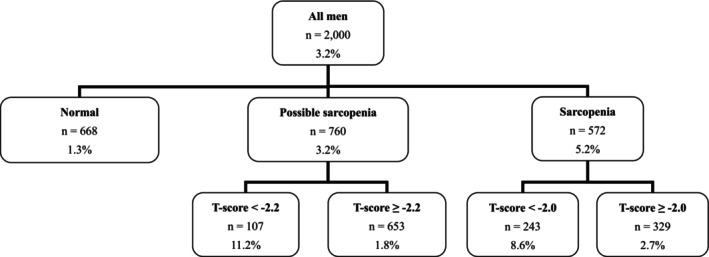
Cart decision tree for identifying cut‐offs of T‐scores to predict hip fractures stratified by sarcopenia status in men. The percentage in the box indicates the percentage of participants with hip fractures over follow‐up period.

**FIGURE 2:**
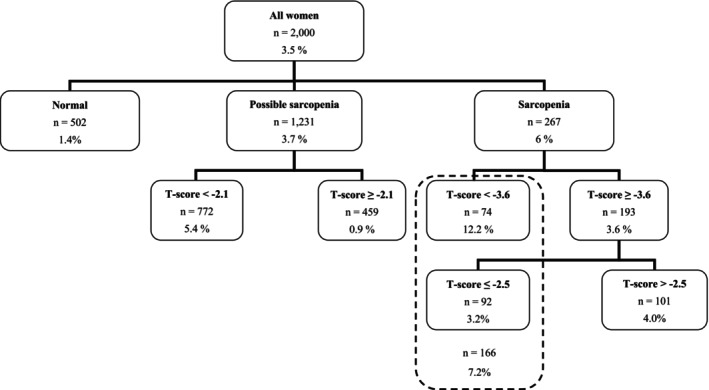
Cart decision tree for identifying cut‐offs of T‐scores to predict hip fractures stratified by sarcopenia status in women. The percentage in the box indicates the percentage of participants with hip fractures over follow‐up period.

From CART analysis, the cut‐off values of T‐score for low BMD in hip fractures prediction among individuals with possible sarcopenia and sarcopenia were −2.2 and −2.0 in men, and −2.1 and −3.6 in women, respectively (Figures 1 and 2). These cut‐offs effectively categorized older individuals into two groups with distinct chances of having hip fractures in men (possible sarcopenia: 11.2% vs. 1.8%; sarcopenia: 8.6% vs. 2.7%) and women (possible sarcopenia: 5.4% vs. 0.9%; sarcopenia: 12.2% vs. 3.6%). In women with sarcopenia, a secondary cut‐off was identified at the T‐score of −2.5, which could separate individuals into two groups with chances of having hip fracture at 7.2% and 4.0%, respectively. The false negative rates were similar between the primary and secondary cut‐offs and using the secondary cut‐off could increase the number of people screened for potential intervention. Therefore, the secondary cut‐off, which is the same as the cut‐off for osteoporosis, will be adopted. The cut‐off values used to define osteosarcopenia were presented in Table 2.

**TABLE 2:** Definition of osteosarcopenia.

Osteosarcopenia definition	Muscle condition		Bone mass condition
Osteosarcopenia_conventional_	Sarcopenia	AND	T‐score < −1.0
Osteosarcopenia_CART_	Possible sarcopenia	AND	Male: T‐score < −2.2 or female: T‐score < −2.1
	Sarcopenia	AND	Male: T‐score < −2.0 or female: T‐score ≤ − 2.5

Abbreviations: Osteosarcopenia_CART_, osteosarcopenia defined according to the cut‐off values of T‐score for different sarcopenia status identified by CART analysis; Osteosarcopenia_conventional_, osteosarcopenia defined as the coexistence of osteopenia/osteoporosis and sarcopenia.

### 3.3 Prediction of Hip Fractures

Results from Cox proportional hazard regression models are shown in Table 3. In men, being osteoporosis, osteosarcopenia_conventional_ and osteosarcopenia_CART_ were all associated with a higher risk of hip fractures even after adjusting FRAX scores (HR = 4.075, 95% CI = 2.45–6.779; HR = 2.494, 95% CI = 1.487–4.184; and HR = 5.232, 95% CI = 3.172–8.631, respectively). In women, being osteoporosis and being osteosarcopenia_CART_ were associated with a higher risk of hip fractures with adjustment of FRAX scores (HR = 2.381, 95% CI = 1.37–4.138, and HR = 3.574, 95% CI = 2.01–6.355, respectively). However, being osteosarcopenia_conventional_ was not significantly associated with hip fractures after adjusting for FRAX scores (HR = 1.501, 95% CI = 0.839–2.687).

**TABLE 3:** Hazard ratios of hip fractures for osteoporosis, osteosarcopenia_conventional_ and osteosarcopenia_CART_ (*n* = 4000).

	Unadjusted model		Model adjusted for FRAX scores		
	HR (95% CI)	*p*	HR (95% CI)	*p*	Uno's C‐index (95% CI)
Men (*n* = 2000)					
Osteoporosis	4.791 (2.891, 7.939)	< 0.001	4.075 (2.45, 6.779)	< 0.001	0.760 (0.697, 0.824)
Osteosarcopenia_conventional_	3.079 (1.876, 5.056)	< 0.001	2.494 (1.487, 4.184)	< 0.001	0.724 (0.659, 0.788)
Osteosarcopenia_CART_	6.155 (3.752, 10.096)	< 0.001	5.232 (3.172, 8.631)	< 0.001	0.774 (0.711, 0.837)
Women (*n* = 2000)					
Osteoporosis	3.027 (1.769, 5.181)	< 0.001	2.381 (1.37, 4.138)	0.002	0.723 (0.665, 0.782)
Osteosarcopenia_conventional_	2.158 (1.234, 3.774)	0.007	1.501 (0.839, 2.687)	0.171	0.731 (0.666, 0.796)
Osteosarcopenia_CART_	4.272 (2.411, 7.569)	< 0.001	3.574 (2.01, 6.355)	< 0.001	0.750 (0.686, 0.815)

Abbreviations: CI, confidence interval; osteosarcopenia_CART_, osteosarcopenia defined according to the cut‐off values of T‐score for different sarcopenia status identified by CART analysis; osteosarcopenia_conventional_, osteosarcopenia defined as the coexistence of osteopenia/osteoporosis and sarcopenia.

Uno's C‐index, NRI and IDI were calculated to assess the hip fracture discriminative and predictive ability of osteoporosis, osteosarcopenia_old_ and osteosarcopenia_CART_. The highest Uno's C‐index was observed for osteosarcopenia_CART_ for hip fractures prediction in both men and women (0.774 and 0.750, respectively, Table 3). Although the difference in C‐index between osteosarcopenia_CART_ and osteoporosis was not significant (men: *p* = 0.407, women: *p* = 0.145), osteosarcopenia_CART_ increased the predictive power by 73.3% in men and 65.2% in women using NRI (*p* < 0.001) and 0.7% in men and 0.5% in women using IDI (*p* < 0.001), suggesting that osteosarcopenia with updated definition outperformed osteoporosis alone in predicting hip fractures in both men and women (Table 4). On the contrary, osteosarcopenia_conventional_ was inferior to osteoporosis alone in predicting hip fractures in both men and women. When compared with osteosarcopenia_conventional_, osteosarcopenia_CART_ not only significantly increased the Uno's C‐index (*p* = 0.033) but also improved the prediction of hip fracture by 76.3% using NRI and 1.8% using IDI in men (*p* < 0.001). Similarly, osteosarcopenia_CART_ also had better predictive ability over osteosarcopenia_conventional_ by 65.3% using NRI and 0.8% using IDI in women (*p* < 0.001).

**TABLE 4:** The hip fracture predictive ability measured by change in Uno's C‐index, IDI and NRI.

	Osteosarcopenia_CART_ vs. osteoporosis (ref.)			Osteosarcopenia_CART_ vs. osteosarcopenia_conventional_ (ref.)			Osteoporosis vs. osteosarcopenia_conventional_ (ref.)		
	∆C‐index (*p*)	NRI (*p*)	IDI (*p*)	∆C‐index (*p*)	NRI (*p*)	IDI (*p*)	∆C‐index (*p*)	NRI (*p*)	IDI (*p*)
Men (*n* = 2000)	0.0138	0.733	0.007	0.0502	0.763	0.018	0.0364	0.548	0.011
	(*p* = 0.407)	(*p* < 0.001)	(*p* < 0.001)	(*p* = 0.033)	(*p* < 0.001)	(*p* < 0.001)	(*p* = 0.134)	(*p* < 0.001)	(*p* < 0.001)
Women (*n* = 2000)	0.0272	0.652	0.005	0.0195	0.653	0.008	−0.0077	0.323	0.003
	(*p* = 0.145)	(*p* < 0.001)	(*p* < 0.001)	(*p* = 0.512)	(*p* < 0.001)	(*p* < 0.001)	(*p* = 0.761)	(*p* = 0.006)	(*p* = 0.002)

Abbreviations: IDI, integrated discrimination improvement; NRI, net reclassification improvement; osteosarcopenia_CART_, osteosarcopenia defined according to the cut‐off values of T‐score for different sarcopenia status identified by CART analysis; osteosarcopenia_conventional_, osteosarcopenia defined as the coexistence of osteopenia/osteoporosis and sarcopenia.

### 3.4 Sensitivity Analysis

Further adjusting for fall history, physical activity levels and number of diseases on top of FRAX scores did not change the associations between osteosarcopenia_CART_ and hip fractures (Model 1, Table S1). Nevertheless, the adjustment of individual components of FRAX resulted in some attenuation of the risk of hip fractures for being osteosarcopenia_CART_, but the associations remained significant (Model 2, Table S1). When excluding participants on osteoporosis medication, being osteosarcopenia_CART_ was still associated with a higher risk of hip fractures and had better predictive ability than being osteoporosis and osteosarcopenia_conventional_ in both older men and women (Tables S2 and S3). When excluding participants with confirmed sarcopenia, both men and women with coexistence of possible sarcopenia and low BMD had an increased risk of hip fractures even with adjustment of FRAX scores (Table S4). The predictive ability of osteosarcopenia_CART_ also outperformed osteoporosis alone with higher Uno's C‐index and improved prediction by using NRI and IDI (Table S4).

## 4 Discussion

To the best of our knowledge, this is the first study to identify sex‐ and sarcopenia‐specific cut‐off of T‐scores for distinguishing individuals at increased risk of hip fractures among community‐dwelling older adults (men with possible sarcopenia: T‐score < −2.2; men with sarcopenia: T‐score < −2.0; women with possible sarcopenia: T‐score < −2.1; women with sarcopenia: T‐score ≤ −2.5). Osteosarcopenia defined using these specific cut‐offs according to sarcopenia status demonstrates a better predictive ability for hip fractures compared with osteosarcopenia defined conventionally (BMD < −1 and sarcopenia) and substantially improves the predictive value over osteoporosis alone. Our findings indicate that including possible sarcopenia and adopting more stringent cut‐offs to define osteosarcopenia could more effectively identify individuals at elevated risk of hip fractures for early prevention or interventions.

From the direct comparisons on the predictive ability for hip fractures, our results revealed the limitation of osteosarcopenia using the conventional definition in hip fracture prediction and demonstrated the predictive value of osteosarcopenia defined by the sex‐ and sarcopenia‐specific cut‐offs of BMD T‐scores. The conventional definition of osteosarcopenia uses one single threshold (T‐scores < −1.0) to define low BMD. With this conventional definition, some previous studies found no significant difference in fracture risks when comparing osteosarcopenia to osteopenia alone [8, 10]. It could be possible that people with higher BMD T‐scores in these studies were classified as having ‘osteosarcopenia’, resulting in an underestimation of the association between osteosarcopenia and fractures. This is supported by a previous meta‐analysis that found that the heterogeneity of the association between osteosarcopenia and fractures disappeared when osteosarcopenia was defined as sarcopenia plus osteoporosis [12]. Our findings have indirectly lent further credence to this postulation. We have identified cut‐offs of T‐scores ranging from −2.5 to −2.0 for having possible sarcopenia and sarcopenia, which were lower than the T‐score of −1.0 and could more effectively identify older adults at elevated risk of hip fractures. Furthermore, osteosarcopenia defined using these CART‐derived cut‐offs of T‐scores improved the predictive ability for hip fractures over osteoporosis alone.

Our findings also highlighted the important role of low muscle strength and low physical performance in the association between osteosarcopenia and hip fractures. When excluding individuals with confirmed sarcopenia, the association between osteosarcopenia_CART_ and hip fractures remained significant and was demonstrated better predictive ability than osteoporosis alone, emphasizing the impact of lower muscle function (low muscle strength and low physical performance) on hip fractures even when muscle mass was preserved. This can be partly explained by the strong associations between low muscle function and increased risk of falls [3]. Additionally, a decline in muscle strength could reduce the mechanical stimuli exerted on the bone, which are essential for osteogenesis [27], thereby increasing the risk of fractures in the event of a fall. With the ageing population, a growing number of older adults may experience possible sarcopenia despite maintaining normal muscle mass. This is because age‐related declines in muscle strength and physical performance tend to occur more rapidly than the loss of muscle mass [28, 29]. As such, including possible sarcopenia in the definition of osteosarcopenia is critically important for hip fractures prevention. Furthermore, the clinical importance of identifying possible sarcopenia is underscored by the limitation of measuring muscle mass by DXA. Although DXA is one of the recommended muscle mass measurements, it is not a direct measure of muscle mass but only provides an estimate of lean mass, which includes non‐muscle components such as soft tissue, fibrotic tissue and water [30]. Additionally, intramuscular fat infiltration impairs muscle quality and muscle strength, accelerating the onset of sarcopenia and increasing fall risks [31]. Future studies are warranted to evaluate the impact of adipose tissue on the association between osteosarcopenia and hip fractures.

Sex difference was observed in the associations between osteosarcopenia and hip fractures in our results. According to the conventional definition, osteosarcopenia was associated with hip fractures in older men but not in older women after adjusting for FRAX scores, suggesting an incremental predictive value of sarcopenia on hip fractures on top of low BMD predominantly for men. This is consistent with the findings in some previous studies [5, 6, 7] and may be explained by the greater decline in muscle mass and greater loss of testosterone in older men than in older women [32, 33]. However, when including possible sarcopenia in the definition, osteosarcopenia became significantly associated with hip fractures in women. This again underscored the importance of muscle strength and physical performance in fracture prediction in both men and women.

Our study advocates early detection and intervention for individuals with osteosarcopenia to mitigate the elevated risk of hip fractures. Osteoporosis treatment is recommended only for individuals with T‐scores ≤ −2.5, or those with T‐scores between −1.0 and −2.5 who have a 10‐year fracture risk of ≥ 3% at the hip or ≥ 20% for any other osteoporotic fracture by FRAX [34]. Although FRAX has included a wide range of clinical risk factors of fractures [35, 36], it does not yet account for the components of sarcopenia. Our study has identified a subgroup of older adults with possible sarcopenia and sarcopenia who are at an increased risk of hip fractures. For these individuals, especially for those who meet our sex‐ and sarcopenia‐specific cut‐offs of T‐scores for osteosarcopenia but do not qualify for osteoporosis treatment according to guideline, non‐pharmaceutical interventions such as exercises and nutritional supplementation should be recommended [37, 38]. Further studies are warranted to validate the cut‐offs of T‐scores identified in our study and to evaluate whether drug treatment in individuals with osteosarcopenia could simultaneously improve bone and muscle condition and attenuate the risk of fractures.

### 4.1 Strength and Limitations

The strength of this study lies in the longitudinal design with a large sample size and the complete record of medication and fractures for over nine years. However, there are several limitations that should be considered when interpreting the results from this study. First, our results might have been biased towards a relatively healthier sample of the community‐dwelling population in Hong Kong who were more educated and physically active; therefore, findings in this study may not be generalized to frail older people or those who are institutionalized. Secondly, sarcopenia status was determined according to AWGS2019 in our study. The findings should be validated in other cohorts using different definitions of sarcopenia. Additionally, the absolute number of hip fractures was relatively small in this study, which has limited our ability to have an internal validation within the study population. An optimistic bias may be introduced in the predictive performance because the CART‐derived cut‐offs of T‐scores were both derived and evaluated in the same analytical sample; therefore, further validation by other external cohorts is also warranted to confirm the generalizability of our findings. Furthermore, our analysis did not formally account for competing risks of deaths, which may overestimate the hazard ratios for hip fracture. However, as we primarily focused on comparing the models within the identical cohort and covariates, our comparative findings are less likely to be affected. Finally, future studies should account for potential time‐varying confounding as we only analysed baseline variables due to the lack of repeated follow‐up assessments.

## 5 Conclusions

Although research on osteosarcopenia is in its infancy, results from this study add to the existing literature that using more stringent cut‐offs for low BMD among those with possible sarcopenia and sarcopenia is warranted to identify older adults at elevated risk of hip fractures. With these sex‐ and sarcopenia‐specific cut‐off values, we found that osteosarcopenia was associated with increased risk of hip fractures with a better predictive ability than osteoporosis alone. Our findings underscore the importance of assessing sarcopenia alongside BMD and the value of osteosarcopenia as a distinct entity for early prevention or intervention to mitigate the increased risk of fractures.

## Funding

This study was supported by the National Institutes of Health (AR049439‐01A1).

## Ethics Statement

The study was approved by the clinical research ethics committee of the Chinese University of Hong Kong, and written informed consents were obtained from all the participants.

## Conflicts of Interest

The authors declare no conflicts of interest.

## Supporting information


**Table S1:** Hazard ratios of hip fractures for osteosarcopenia_CART_ among older men and women (*n* = 4000).

## References

[1] O. Johnell and J. A. Kanis , “An Estimate of the Worldwide Prevalence and Disability Associated With Osteoporotic Fractures,” Osteoporosis International 17 (2006): 1726–1733. 16983459 10.1007/s00198-006-0172-4

[2] K. E. Ensrud , “Epidemiology of Fracture Risk With Advancing Age,” Journals of Gerontology. Series A, Biological Sciences and Medical Sciences 68 (2013): 1236–1242. 23833201 10.1093/gerona/glt092

[3] S. S. Y. Yeung , E. M. Reijnierse , V. K. Pham , et al., “Sarcopenia and Its Association With Falls and Fractures in Older Adults: A Systematic Review and Meta‐Analysis,” Journal of Cachexia, Sarcopenia and Muscle 10 (2019): 485–500. 30993881 10.1002/jcsm.12411 PMC6596401

[4] B. Kirk , J. Zanker , and G. Duque , “Osteosarcopenia: Epidemiology, Diagnosis, and Treatment—Facts and Numbers,” Journal of Cachexia, Sarcopenia and Muscle 11 (2020): 609–618. 32202056 10.1002/jcsm.12567 PMC7296259

[5] D. Chalhoub , P. M. Cawthon , K. E. Ensrud , et al., “Risk of Nonspine Fractures in Older Adults With Sarcopenia, Low Bone Mass, or Both,” Journal of the American Geriatrics Society 63 (2015): 1733–1740. 26310882 10.1111/jgs.13605 PMC4625906

[6] A. Lee , C. McArthur , G. Ioannidis , et al., “Associations Between Osteosarcopenia and Falls, Fractures, and Frailty in Older Adults: Results From the Canadian Longitudinal Study on Aging (CLSA),” Journal of the American Medical Directors Association 25 (2024): 167–176. 37925161 10.1016/j.jamda.2023.09.027

[7] R. Yu , J. Leung , and J. Woo , “Incremental Predictive Value of Sarcopenia for Incident Fracture in an Elderly Chinese Cohort: Results From the Osteoporotic Fractures in Men (MrOs) Study,” Journal of the American Medical Directors Association 15 (2014): 551–558. 24703927 10.1016/j.jamda.2014.02.005

[8] K. Trajanoska , J. D. Schoufour , S. K. L. Darweesh , et al., “Sarcopenia and Its Clinical Correlates in the General Population: The Rotterdam Study,” Journal of Bone and Mineral Research 33 (2018): 1209–1218. 29502340 10.1002/jbmr.3416

[9] D. Scott , M. Seibel , R. Cumming , et al., “Does Combined Osteopenia/Osteoporosis and Sarcopenia Confer Greater Risk of Falls and Fracture Than Either Condition Alone in Older Men? The Concord Health and Ageing in Men Project,” Journals of Gerontology: Series A 74 (2019): 827–834. 10.1093/gerona/gly162 30032209

[10] S. Balogun , T. Winzenberg , K. Wills , et al., “Prospective Associations of Osteosarcopenia and Osteodynapenia With Incident Fracture and Mortality Over 10 Years in Community‐Dwelling Older Adults,” Archives of Gerontology and Geriatrics 82 (2019): 67–73. 30716680 10.1016/j.archger.2019.01.015

[11] F. Salech , C. Marquez , L. Lera , B. Angel , R. Saguez , and C. Albala , “Osteosarcopenia Predicts Falls, Fractures, and Mortality in Chilean Community‐Dwelling Older Adults,” Journal of the American Medical Directors Association 22 (2021): 853–858. 32921573 10.1016/j.jamda.2020.07.032

[12] Z. Teng , Y. Zhu , Y. Teng , et al., “The Analysis of Osteosarcopenia as a Risk Factor for Fractures, Mortality, and Falls,” Osteoporosis International 32 (2021): 2173–2183. 33877382 10.1007/s00198-021-05963-x

[13] B. Kirk , A. Al Saedi , and G. Duque , “Osteosarcopenia: A Case of Geroscience,” Aging Med (Milton) 2 (2019): 147–156. 31942528 10.1002/agm2.12080 PMC6880711

[14] F. B. Pereira , A. F. Leite , and A. P. de Paula , “Relationship Between Pre‐Sarcopenia, Sarcopenia and Bone Mineral Density in Elderly Men,” Archives of Endocrinology and Metabolism 59 (2015): 59–65. 25926116 10.1590/2359-3997000000011

[15] D. Scott , J. Johansson , L. B. McMillan , et al., “Associations of Sarcopenia and Its Components With Bone Structure and Incident Falls in Swedish Older Adults,” Calcified Tissue International 105 (2019): 26–36. 30899995 10.1007/s00223-019-00540-1

[16] P. Szulc , T. J. Beck , F. Marchand , and P. D. Delmas , “Low Skeletal Muscle Mass Is Associated With Poor Structural Parameters of Bone and Impaired Balance in Elderly Men—The MINOS Study,” Journal of Bone and Mineral Research 20 (2005): 721–729. 15824844 10.1359/JBMR.041230

[17] M. E. Tinetti , M. Speechley , and S. F. Ginter , “Risk Factors for Falls Among Elderly Persons Living in the Community,” New England Journal of Medicine 319 (1988): 1701–1707. 3205267 10.1056/NEJM198812293192604

[18] W. Sepúlveda‐Loyola , S. Phu , E. Bani Hassan , et al., “The Joint Occurrence of Osteoporosis and Sarcopenia (Osteosarcopenia): Definitions and Characteristics,” Journal of the American Medical Directors Association 21 (2020): 220–225. 31669290 10.1016/j.jamda.2019.09.005

[19] S. Balogun , T. Winzenberg , K. Wills , et al., “Prospective Associations of Low Muscle Mass and Function With 10‐Year Falls Risk, Incident Fracture and Mortality in Community‐Dwelling Older Adults,” Journal of Nutrition, Health & Aging 21 (2017): 843–848. 10.1007/s12603-016-0843-6 PMC12878221 28717816

[20] L. K. Chen , J. Woo , P. Assantachai , et al., “Asian Working Group for Sarcopenia: 2019 Consensus Update on Sarcopenia Diagnosis and Treatment,” Journal of the American Medical Directors Association 21 (2020): 300–307. 32033882 10.1016/j.jamda.2019.12.012

[21] S. B. Heymsfield , R. Smith , M. Aulet , et al., “Appendicular Skeletal Muscle Mass: Measurement by Dual‐Photon Absorptiometry,” American Journal of Clinical Nutrition 52 (1990): 214–218. 2375286 10.1093/ajcn/52.2.214

[22] “World Health Organization Collaborating Centre for Metabolic Bone Diseases at the University of Sheffield,” UK FRAX calculation tool (Hong Kong), accessed April 11, 2024, https://frax.shef.ac.uk/FRAX/tool.aspx?country=20.

[23] R. A. Washburn , K. W. Smith , A. M. Jette , and C. A. Janney , “The Physical Activity Scale for the Elderly (PASE): Development and Evaluation,” Journal of Clinical Epidemiology 46 (1993): 153–162. 8437031 10.1016/0895-4356(93)90053-4

[24] L. Breiman , J. Friedman , C. J. Stone , et al., Classification and Regression Trees (Wadsworth International Group, 1984).

[25] H. Uno , T. Cai , M. J. Pencina , R. B. D'Agostino , and L. J. Wei , “On the C‐Statistics for Evaluating Overall Adequacy of Risk Prediction Procedures With Censored Survival Data,” Statistics in Medicine 30 (2011): 1105–1117. 21484848 10.1002/sim.4154 PMC3079915

[26] M. J. Pencina , R. B. D'Agostino , and E. W. Steyerberg , “Extensions of Net Reclassification Improvement Calculations to Measure Usefulness of New Biomarkers,” Statistics in Medicine 30 (2010): 11–21. 21204120 10.1002/sim.4085 PMC3341973

[27] P. Christen , K. Ito , R. Ellouz , et al., “Bone Remodelling in Humans is Load‐Driven but Not Lazy,” Nature Communications 5 (2014): 1–5. 10.1038/ncomms5855 25209333

[28] T. W. Auyeung , S. W. J. Lee , J. Leung , T. Kwok , and J. Woo , “Age‐Associated Decline of Muscle Mass, Grip Strength and Gait Speed: A 4‐Year Longitudinal Study of 3018 Community‐Dwelling Older Chinese,” Geriatrics & Gerontology International 14 (2014): 76–84. 24450564 10.1111/ggi.12213

[29] L. dos Santos , E. S. Cyrino , M. Antunes , D. A. Santos , and L. B. Sardinha , “Sarcopenia and Physical Independence in Older Adults: The Independent and Synergic Role of Muscle Mass and Muscle Function,” Journal of Cachexia, Sarcopenia and Muscle 8 (2017): 245–250. 27897417 10.1002/jcsm.12160 PMC5377449

[30] P. M. Cawthon , K. E. Peters , S. R. Cummings , et al., “Association Between Muscle Mass Determined by D3‐Creatine Dilution and Incident Fractures in a Prospective Cohort Study of Older Men,” Journal of Bone and Mineral Research 37 (2022): 1213–1220. 35253257 10.1002/jbmr.4505 PMC9283198

[31] M. Ramírez Torres , R. E. Ruiz Valenzuela , J. Esparza‐Romero , M. T. López Teros , and H. Alemán‐Mateo , “The Fat Mass Index, not the Fat‐Free Mass Index, Is Associated With Impaired Physical Performance in Older Adult Subjects: Evidence From a Cross‐Sectional Study,” Clinical Nutrition 38 (2019): 877–882. 29501367 10.1016/j.clnu.2018.02.013

[32] I. Janssen , S. B. Heymsfield , Z. M. Wang , and R. Ross , “Skeletal Muscle Mass and Distribution in 468 Men and Women Aged 18–88 Yr,” Journal of Applied Physiology 89 (2000): 81–88. 10904038 10.1152/jappl.2000.89.1.81

[33] T. W. Auyeung , J. S. W. Lee , T. Kwok , et al., “Testosterone but Not Estradiol Level is Positively Related to Muscle Strength and Physical Performance Independent of Muscle Mass: A Cross‐Sectional Study in 1489 Older Men,” European Journal of Endocrinology 164 (2011): 811–817. 21346095 10.1530/EJE-10-0952

[34] F. Cosman , S. J. de Beur , M. S. LeBoff , et al., “Clinician's Guide to Prevention and Treatment of Osteoporosis,” Osteoporosis International 25 (2014): 2359–2381. 25182228 10.1007/s00198-014-2794-2 PMC4176573

[35] J. A. Kanis , O. Johnell , A. Oden , H. Johansson , and E. McCloskey , “FRAX and the Assessment of Fracture Probability in Men and Women From the UK,” Osteoporosis International 19 (2008): 385–397. 18292978 10.1007/s00198-007-0543-5 PMC2267485

[36] L. Vandenput , H. Johansson , E. V. McCloskey , et al., “Update of the Fracture Risk Prediction Tool FRAX: A Systematic Review of Potential Cohorts and Analysis Plan,” Osteoporosis International 33 (2022): 2103–2136. 35639106 10.1007/s00198-022-06435-6

[37] R. M. Daly , J. Gianoudis , M. E. Kersh , et al., “Effects of a 12‐Month Supervised, Community‐Based, Multimodal Exercise Program Followed by a 6‐Month Research‐to‐Practice Transition on Bone Mineral Density, Trabecular Microarchitecture, and Physical Function in Older Adults: A Randomized Controlled Trial,” Journal of Bone and Mineral Research 35 (2020): 419–429. 31498937 10.1002/jbmr.3865

[38] L. Y. Zhu , R. Chan , T. Kwok , K. C. C. Cheng , A. Ha , and J. Woo , “Effects of Exercise and Nutrition Supplementation in Community‐Dwelling Older Chinese People With Sarcopenia: A Randomized Controlled Trial,” Age and Ageing 48 (2019): 220–228. 30462162 10.1093/ageing/afy179

