# Supplementary material for: Improving Hip Fracture Prediction by Using Sarcopenia‐Specific Cut‐Offs of T‐Scores for Osteosarcopenia: A Prospective Cohort Study
Source: J Cachexia Sarcopenia Muscle. 2026 Jul 15;17(4):e70348. doi: 10.1002/jcsm.70348 (PMC13371577; doi:10.1002/jcsm.70348)
Supplement: Supplementary file 1 — Table S1: Hazard ratios of hip fractures for osteosarcopeniaCART among older men and women (n = 4000). [file JCSM-17-e70348-s001.docx]

Supplementary Table 1. Hazard ratios of hip fractures for osteosarcopenia_CART_ among older men and women (n=4,000).

|  | **Model 1** | |  | **Model 2** | |
| --- | --- | --- | --- | --- | --- |
|  | **HR (95% CI)** | ***p* value** |  | **HR (95% CI)** | ***p* value** |
| Men (n=2,000) | 5.22 (3.164, 8.612) | <0.001 |  | 4.218 (2.315, 7.687) | <0.001 |
| Women (n=2,000) | 3.528 (1.981, 6.284) | <0.001 |  | 2.777 (1.437, 5.365) | 0.002 |

Model 1: adjusted for FRAX scores, history of falls, physical activity, and number of diseases.

Model 2: adjusted for individual FRAX components including age, sex, BMI, previous history of fracture, parental history of hip fracture, current smoking, at least 3 alcoholic beverages per day, glucocorticoids use, rheumatoid arthritis, secondary osteoporosis.

Osteosarcopenia_CART_, osteosarcopenia defined according to the cutoff values of T-score for different sarcopenia status identified by CART analysis.

**Supplementary Table 2. Hazard ratios of hip fractures for osteoporosis, osteosarcopenia_conventional_, and osteosarcopenia_CART_ among participants without osteoporosis medication (N=3,704).**

|  | **Unadjusted model** | |  | **Model adjusted for FRAX score** | |  |
| --- | --- | --- | --- | --- | --- | --- |
|  | **HR (95% CI)** | ***p* value** |  | **HR (95% CI)** | ***p* value** | **Uno’s C-index (95%CI)** |
| Men (n=1,957) |  |  |  |  |  |  |
| Osteoporosis | 5.074 (3.042, 8.462) | <0.001 |  | 4.271 (2.55, 7.153) | <0.001 | 0.763 (0.707, 0.820) |
| Osteosarcopenia_conventional_ | 3.194 (1.938, 5.261) | <0.001 |  | 2.576 (1.53, 4.34) | <0.001 | 0.728 (0.668, 0.789) |
| Osteosarcopenia_CART_ | 6.246 (3.794, 10.28) | <0.001 |  | 5.282 (3.19, 8.745) | <0.001 | 0.775 (0.714, 0.836) |
| Women (n=1,747) |  |  |  |  |  |  |
| Osteoporosis | 3.013 (1.741, 5.214) | <0.001 |  | 2.332 (1.322, 4.112) | 0.003 | 0.723 (0.669, 0.777) |
| Osteosarcopenia_conventional_ | 2.437 (1.38, 4.304) | 0.002 |  | 1.673 (0.922, 3.036) | 0.09 | 0.732 (0.674, 0.790) |
| Osteosarcopenia_CART_ | 4.468 (2.432, 8.103) | <0.001 |  | 3.708 (2.036, 6.754) | <0.001 | 0.754 (0.703, 0.804) |

Osteosarcopenia_conventional_, osteosarcopenia defined as the coexistence of osteopenia/osteoporosis and sarcopenia; Osteosarcopenia_CART_, osteosarcopenia defined according to the cutoff values of T-score for different sarcopenia status identified by CART analysis.

**Supplementary Table 3. The hip fracture predictive ability measured by change in Uno’s C-index, IDI, and NRI among participants without osteoporosis medication (N=3,704).**

|  | **Osteosarcopenia_CART_**  **vs.**  **Osteoporosis (ref.)** | | |  | **Osteosarcopenia_CART_**  **vs.**  **Osteosarcopenia_conventional_ (ref.)** | | |  | **Osteoporosis**  **vs.**  **Osteosarcopenia_conventional_ (ref.)** | | |
| --- | --- | --- | --- | --- | --- | --- | --- | --- | --- | --- | --- |
|  | **∆C-index**  **(*p* value)** | **NRI**  **(*p* value)** | **IDI**  **(*p* value)** |  | **∆C-index**  **(*p* value)** | **NRI**  **(*p* value)** | **IDI**  **(*p* value)** |  | **∆C-index**  **(*p* value)** | **NRI**  **(*p* value)** | **IDI**  **(*p* value)** |
| Men (n=1,957) | 0.011 (*p*=0.560) | 0.516 (*p*<0.001) | 0.007 (*p*<0.001) |  | 0.047 (*p*=0.023) | 0.758 (*p*<0.001) | 0.018 (*p*<0.001) |  | 0.035 (*p*=0.144) | 0.518 (*p*<0.001) | 0.011 (*p*<0.001) |
|  |  |  |  |  |  |  |  |  |  |  |  |
| Women  (n=1,747) | 0.031 (*p*=0.100) | 0.683 (*p*<0.001) | 0.007 (*p*<0.001) |  | 0.022 (*p*=0.425) | 0.684 (*p*<0.001) | 0.008 (*p*<0.001) |  | -0.009 (*p*=0.708) | 0.291 (*p*=0.020) | 0.002 (*p*=0.002) |

Osteosarcopenia_conventional_, osteosarcopenia defined as the coexistence of osteopenia/osteoporosis and sarcopenia; Osteosarcopenia_CART_, osteosarcopenia defined according to the cutoff values of T-score for different sarcopenia status identified by CART analysis; NRI, net reclassiﬁcation improvement; IDI, integrated discrimination improvement.

Supplementary Table 4: Hazard ratios of hip fractures for osteoporosis and osteosarcopenia_CART_ among participants without confirmed sarcopenia (n=3,161).

|  | **Unadjusted model** | |  | **Model adjusted for FRAX score** | |  |  |  |  |
| --- | --- | --- | --- | --- | --- | --- | --- | --- | --- |
|  | **HR (95% CI)** | ***p* value** |  | **HR (95% CI)** | ***p* value** | **Uno’s C-index (95%CI)** | **∆C-index**  **(*p* value)** | **NRI**  **(*p* value)** | **IDI**  **(*p* value)** |
| Men (n=1,428) |  |  |  |  |  |  |  |  |  |
| Osteoporosis | 5.664 (2.787, 11.514) | <0.001 |  | 4.874 (2.388, 9.946) | <0.001 | 0.745 (0.650, 0.839) | 0.015 (*p*=0.604) | 0.651 (*p*<0.001) | 0.012  (*p*=0.074) |
| Osteosarcopenia_CART_ | 8.257 (4.061, 16.79) | <0.001 |  | 7.204 (3.532, 14.692) | <0.001 | 0.760 (0.664, 0.855) |  |  |  |
| Women (n=1,733) |  |  |  |  |  |  |  |  |  |
| Osteoporosis | 3.207 (1.741, 5.906) | <0.001 |  | 2.563 (1.371, 4.791) | 0.003 | 0.736 (0.673, 0.799) | 0.034 (*p*=0.157) | 0.717 (*p*<0.001) | 0.009  (*p*=0.005) |
| Osteosarcopenia_CART_ | 4.962 (2.554, 9.637) | <0.001 |  | 4.418 (2.274, 8.584) | <0.001 | 0.769 (0.709, 0.830) |  |  |  |

Osteosarcopenia_CART_, osteosarcopenia defined according to the cutoff values of T-score for different sarcopenia status identified by CART analysis; NRI, net reclassiﬁcation improvement; IDI, integrated discrimination improvement.
